# Airport microgrid control using integral-of-absolute-error having high renewable penetration

**DOI:** 10.1038/s41598-025-04820-9

**Published:** 2025-07-02

**Authors:** Sameer Singh, V. P. Singh, A. Mathur, T. K. Bashishtha, Sanjeevikumar Padmanaban, T. Varshney

**Affiliations:** 1 Electrical Engineering Department, Malaviya National Institute of Technology, Jaipur, 302017 Rajasthan India; 2 Department of Electrical Engineering, IT and Cybernetics, University of South-Eastern Norway, 3918 Porsgrunn, Norway; 3 EECE, SSET, Sharda University, GB Nagar, 201306 Uttar Pradesh India

**Keywords:** Airport microgrid, PID Controller, Frequency stability, Error index, Engineering, Aerospace engineering

## Abstract

The serious concern about the continuous depletion of fossil fuels and their environmental impact has drawn the focus of researchers worldwide, towards the renewable energy sector. Renewable resources are being penetrated into microgrids on a larger scale in order to manage sustainable financial and environmental viability. Increased penetration of renewable resources has increased the operational challenges associated with it. Moreover, the stochastic nature of renewable resources with the combined effect of load disturbances, causes frequency deviation at a considerable scale. Minimization of frequency deviation is a crucial task for maintaining the stability of airport microgrid (AP${\mu }G_{d}$). To deal with the aforementioned operational challenges, in this article, frequency deviation is managed by designing the PID controller employing integral absolute error (IAE) for an AP${\mu }G_{d}$ system. Firstly, the overall transfer function (OATrFn) for AP${\mu }G_{d}$ system is obtained by modeling and combining each component. For easier and more efficient analytical study cum controller design, the first order plus delay time (FOPDT) model is obtained for the AP${\mu }G_{d}$ system. A detailed analysis in terms of frequency deviation and controller effort is carried out for AP${\mu }G_{d}$ system with and without a PID controller to validate the impact of a PID controller in maintaining the frequency stability of AP${\mu }G_{d}$ system. Further, a comparative study for the same system is performed considering the integral time absolute error (ITAE) as a main design criterion. Tabular data and various plots validate the superiority of IAE driven PID controller over ITAE-PID controller to maintain frequency stability. Furthermore, a bar plot is plotted to provide a comparative analysis among various error indices in the form of frequency deviations.

## Introduction

Matching the generation with the demands keeping minimum losses is a key requirement for the design and implementation of an efficient power system. In a conventional system, electricity is generated and transmitted for a longer distance to meet the load demand at the consumer terminal. Long-distance transmission causes transmission losses to a higher degree. Apart from that, long-distance transmission also consists of various drawbacks such as several points of control throughout the distance, difficulty in identifying the fault locations, voltage drop, power theft, etc. Such drawbacks are overcome to a great level by replacing centralized generation with distributed generating stations. Incorporating distributed small-scale generation, based on locally available resources to meet the load in the locality, facilitated with a suitable control structure, is interpreted as a microgrid (${\mu }G_{d}$). The prime advantage of a ${\mu }G_{d}$ system is its capability to operate in two modes which are categorized as grid-connected and standalone mode. Depending on the scenario, ${\mu }G_{d}$ can isolate itself and operate autonomously to satisfy the local load demand efficiently. In literature^1^, authors presented a critical review on various ${\mu }G_{d}$ architecture. Micallef et al. ^2^, discussed the detailed analysis for the diverse application of ${\mu }G_{d}$ such as airport (AP) ${\mu }G_{d}$, shipboard (SP) ${\mu }G_{d}$, space (Sc) ${\mu }G_{d}$, and port (Pt) ${\mu }G_{d}$. The contribution also involves the identification of merits for operating the ${\mu }G_{d}$ during grid-connected and islanded modes. Further, several contributions are reported in the literature for the modeling, analysis, and control of airport microgrids (AP${\mu }G_{d}$).

In^3^, strategy for optimum scheduling of heterogeneous energy storage is developed for AP${\mu }G_{d}$. Wang et al. ^4^, proposed a scheduling model for mobile energy storage system for AC-DC hybrid microgrids by considering mode of operation and time of use costing. The authors further demonstrated the effectiveness of a simplified airport system and confirmed its applicability through the practical analysis of a real airport. Moreover, Talebi et al.,^5^ An AP${\mu }G_{d}$ microgrid model is proposed for exploring the utilization of renewable resources in order to electrify the small rurally located airports, while managing renewable capacity, size of the system and storage. Authors have claimed the efficacy of their contribution through the case study demonstration at Aydin airport accounting constraints of both airport and system. Contribution for improving the resiliency of a grid connected AP${\mu }G_{d}$ during outages, is presented in literature by Alruwali et al.^6^ Moreover, Guo et al. performed an analytical study to analyze the frequency response by integrating the aircraft to the grid. Authors have executed strategy for energy dispach on hourly basis incorporating seasonal scheduling of flights. In order to perform techno-economic analysis, Zhao et al. have carried out technical analysis based on economic concerns considering numerous distributed generating units, storage components, integrated electric vehicles and aircraft to the airport energy supply system. Moreover economical analysis, Ajayi et al. ^7^, proposed the aircraft taxing conceptual model as such energy as service and managed the power flow across grid and renewable resources in support of vehicle to grid (V2G) capability enhancement. However, a comparative analysis for the integrated hybrid renewable energy system in various configurations is performed in order to determine the energy cost, renewable penetration and fuel consumption at the Mwanza airport by Minja et al. ^8^. Further, Kilmi et al.,^9^, have presented a case study for the improvement of power supply during off-grid. Moreover, an energy management system is proposed for a hangar microgrid in^10^. Authors have incorporated uncertainties and managed the resource dispatch using model predictive control. A study for designing and planning for the hybrid renewable energy system is discussed in^11^. The social and economic benefits are highlighted based on multi-criteria decision-making by the authors.

**Table 1:** Summary of studies on airport microgrid systems and their contributions.

References	Author(s)	WDG	PV	DOG	EV	AC	BBES	FC	Contribution
^6^	Alruwaili et al.		$\checkmark$	$\checkmark$			$\checkmark$		Annual cost reduction by optimizing microgrid dispatching and sizing.
^12^	Song et al.	$\checkmark$	$\checkmark$	$\checkmark$			$\checkmark$	$\checkmark$	Developed a dispatch model for airport microgrids.
^13^	Liaw et al.				$\checkmark$	$\checkmark$	$\checkmark$	$\checkmark$	Designed a bipolar DC microgrid for airports.
^8^	Minja et al.	$\checkmark$	$\checkmark$	$\checkmark$			$\checkmark$		Simulated a hybrid energy system for Mwanza Airport.
^5^	Talebi et al.	$\checkmark$	$\checkmark$	$\checkmark$			$\checkmark$		Proposed a high-level rural airport microgrid model.
^14^	Abubakr et al.		$\checkmark$	$\checkmark$	$\checkmark$		$\checkmark$		Enhanced frequency stability with control strategies for microgrids.
^15^	Yildirim et al.	$\checkmark$	$\checkmark$				$\checkmark$	$\checkmark$	Designed an advanced controller for islanded microgrid load-frequency control.
^4^	Liu et al.						$\checkmark$		Proposed a mobile energy storage system to enhance environmental and economic benefits.
^16^	Zhou et al.		$\checkmark$				$\checkmark$	$\checkmark$	Proposed an energy system integrating large-scale hydrogen resources.
^10^	Verdugo et al.		$\checkmark$				$\checkmark$		Developed a hangar microgrid model for thermoelectric grid resource management.
^17^	Xiang et al.		$\checkmark$		$\checkmark$		$\checkmark$	$\checkmark$	Conducted a techno-economic analysis of airport energy systems.
^18^	Aff et al.	$\checkmark$	$\checkmark$	$\checkmark$			$\checkmark$	$\checkmark$	Implemented control-based damping to reduce frequency fluctuations in hybrid microgrids.
^19^	Tu et al.	$\checkmark$	$\checkmark$				$\checkmark$	$\checkmark$	Proposed optimal reset control for hybrid microgrid systems.

An airport microgrid architecture should consists of various distributed resources along with control schemes to manage the supply and demands efficiently. Several architectures are so far reported in the literature in order to develop the airport microgrid for uninterrupted efficient electrification^6,8,12,13^. In Alruwaili et al.^6^ evaluated the study for a grid-connected airport microgrid incorporating energy storage, solar PV, and diesel generators for backup supply during power outages. The author further contributed for the optimization of the dispatching and sizing of microgrids to reduce annual costs. Furthermore, Song et al.^12^, developed a dispatch model for airport microgrid involving PV, wind mills, diesel engine generator, fuel cell and battery storage. Author has utilized an algorithm to increase the optimization accuracy as deviated operating cost. Moreover, Liaw et al.^13^ developed a bipolar DC microgrid for airport electrification integrated with the utility grid. Authors have involved the electric vehicle and aircraft in the bidirectional mode of operation in the architecture, along with PV and fuel cells as storage to maintain the DC bus voltage of the airport microgrid. In Minja et al.^8^ modelled and simulated a hybrid energy system (renewable and non renewable) for Mwanza international airport Tanzania. For the airport energy system architecture, author has utilized wind energy, diesel generator, PV and battery storage to design and simulate hybrid energy system. In Liaw et al.^13^ discussed the airport DC microgrid operation incorporating electric vehicle (EVhcle) and Aircraft (AcrFt). As per the resources available in surroundings, a fundamental structure of a microgrid may exhibit various renewable-based generation such as wind-driven generators (WDG), solar-dependent photovoltaic cells (SDPVC), bio-energy resources (BES), etc. Moreover, renewable resources, for backup and mitigation of instant supply fluctuation, ${\mu }G_{d}$ system incorporates diesel-operated generator (DOG) and battery-based energy storage (BBES) equipment, etc.

When various generating and storage units are integrated into AP${\mu }G_{d}$, the system dynamics become more complex, making stability and control difficult. To guarantee effective operation under a range of load and generation situations, it is imperative to design a PID controller for such systems due to its simplicity, reliability, and ease of implementation. Moreover, optimal transient performance and error minimization are guaranteed when PID tuning uses performance indices like ITAE and IAE. The necessity to close the gap between realistic controller design and complicated system modeling to enable reliable and efficient management of AP${\mu }G_{d}$ systems.

In this contribution, PID control scheme is implemented based on ITAE and IAE indices for the AP${\mu }G_{d}$ system . Firstly, an architecture is designed incorporating various generating and storage resources. Each components of the architecture is modelled in the form of first order transfer function (FOTrFn). Further, contribution continued in order to figure out the overall (OATrFn) by combining the each component of the AP${\mu }G_{d}$ system modelled in FOTrFn. The OATrFn of AP${\mu }G_{d}$ system is of higher order (HiOr). Being of a HiOr, OATrFn posses some drawbacks such as analytical study, controller design, etc. To overcome these drawbacks, an approximated first order plus delay time (FOPDT) model is obtained for the OATrFn of higher order. Further, controller implementation is accounted by utilizing ITAE and IAE indices, and gains of the PID controller are tuned^20,21^. Effectiveness of the PID controller is analyzed through the frequency response plotted before and after the implementation of the controller. Tabular data for time-domain specifications are also provided in support of the utilized approach.

- The mathematical modeling of AP${\mu }G_{d}$ system in terms of overall transfer function (OATrFn) is derived by combining the transfer functions of all individual components, including generating and storage resources.
- A first-order plus delay time (FOPDT) model is developed for AP${\mu }G_{d}$ utilizing OATrFn, to design efficient PID control design.
- Utilized an efficient tuning methodology for PID controller considering frequency deviation of AP${\mu }G_{d}$ with Integral Absolute Error (IAE), for deriving controller gains.
- The efficacy of PID controller is established in minimizing frequency deviations through comprehensive analysis in terms of time-domain specifications and frequency responses of AP${\mu }G_{d}$.
- A detailed comparative study highlights the effectiveness of IAE and ITAE-based PID controllers, showcasing their respective advantages in achieving system stability and optimal performance.

This paper is organized in the following structure. Architecture for AP${\mu }G_{d}$ system with corresponding mathematical representation of components and generalized OATrFn, are discussed in section II. FOPDT model for generalized OATrFn of AP${\mu }G_{d}$ is discussed in section III. In section IV, PID controller design and implementation in AP${\mu }G_{d}$ is described. Validation of FOPDT model with AP${\mu }G_{d}$ model and frequency regulation are explained in section V. Section VI dealt with the results and discussion by exploring the role of PID controller in minimizing frequency deviations of AP${\mu }G_{d}$. Finally, conclusion of the presented work with possible future work is discussed in section VII.

## Architecture of airport microgrid

**Fig. 1:**
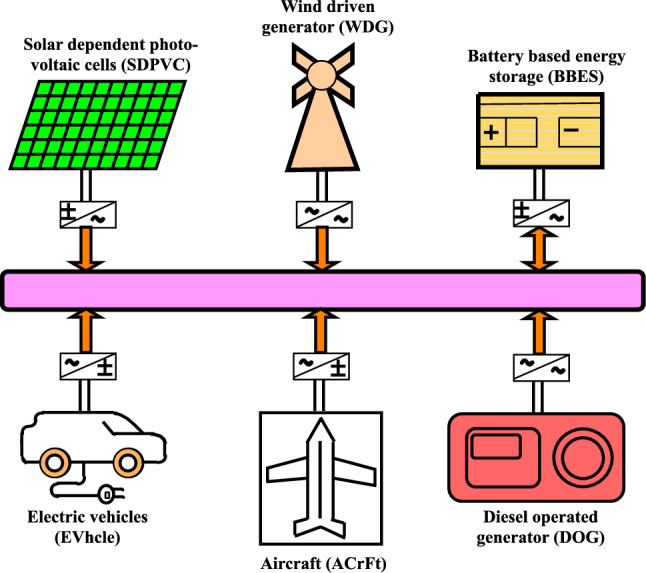
Architecture of AP${\mu }G_{d}$ System.

The AP${\mu }G_{d}$ architecture considered in this work, is depicted in Fig. 1. Each generating and storage unit of the AP${\mu }G_{d}$ are interconnected for the optimum supply to the load via power converters. Generating and storage units are consists of SDPVC, WDG, DOG, and BBES. Among the available generating units SDPVC and WDG are weather dependent, thus stochastic in nature. Further, due to variation in sunlight intensity and speed of wind, the power output of SDPVC and WDG are variable. On the other hand, load is also irregular with respect to time. The uneven load and variable power output from SDPVC and WDG, combindly create disturbances, causing mismatch of generation and demand. The mismatching of supply and demands leads to the frequency fluctuation which needs to be maintained as minimum as possible. In Fig. 1, BBES is provided for immediate storage. When generation exceeds the demands, excess power gets stored in the BBES and supplied power during power deficiency. Moreover, to the aforementioned generating and storage units, the architecture of the AP${\mu }G_{d}$ system is specialized with the integration of aircraft (ACrFt) and electric vehicles (EVhcle), which are present at the airport. ACrFt and EVhcle are utilized as the storage resources as per the demands and state of charge (SOC) of the battery of ACrFt and EVhcle. For each unit of generation and storage, modeling is done in the form of FOTrFn. The mathematical modeling of AP${\mu }G_{d}$ components is described in (1)-(7).

### Solar dependent photovoltaic cells (SDPVC)

Solar panels are used to generate electricity by utilizing the heat and intensity of the sunlight. The transfer function for the solar-dependent photo voltaic cell is given in (1).

1 $$\begin{aligned} H_{SDPVC}(s)=\frac{G_{SDPVC}}{1+sT_{SDPVC}} \end{aligned}$$

As given in (1), time constant and gain representation are done by $T_{SDPVC}$ and $G_{SDPVC}$, respectively.

### Wind driven generator (WDG)

Due to the kinetic energy of blowing wind, force is exerted on the blade of wind mills which causes spinning of rotor. Rotor is mechanically coupled with the generator for producing electricity. The transfer function for the wind driven generator is written in first order in (2).

2 $$\begin{aligned} H_{WDG}(s)=\frac{G_{WDG}}{1+sT_{WDG}} \end{aligned}$$

As depicted in (2), $G_{WDG}$ and $T_{WDG}$ are used for denoting the gain and time constant, respectively.

### Aircraft (ACrFt)

At the airport premises, available aircrafts are utilized as an storage element. Modeling of aircraft in first order is given in (3).

3 $$\begin{aligned} H_{ACrFt}(s)=\frac{G_{ACrFt}}{1+sT_{ACrFt}} \end{aligned}$$

In (3), gain and time constant for ACrFt is depicted as $$ G_{ACrFt}$$ and $$T_{ACrFt}$$, respectively.

### Electric vehicles (EVhcle)

The parking area of the airport filled with electric vehicles (EVhcle), is facilitated with plugin to grid facilities and utilized as the energy storage. Transfer function-based mathematical modeling of the EVhcle is depicted in (4).

4 $$\begin{aligned} H_{EVhcle}(s)=\frac{G_{EVhcle}}{1+sT_{EVhcle}} \end{aligned}$$

In (4), gain is represented as $G_{EVhcle}$, whereas time constant is denoted by $T_{EVhcle}$.

### Diesel operated generator (DOG)

Diesel operated generator is for providing the backup supply to the load. DOG is modelled by combining the mathematical model of generator and governor. The transfer function for the generator and governor is given in (5).

5 $$\begin{aligned} H_{DOG}(s)=\left( \frac{G_{Gen}}{1+sT_{Gen}}\right) \left( \frac{G_{Gov}}{1+sT_{Gov}}\right) \end{aligned}$$

In (5), for generator, $G_{Gen}$ and $T_{Gen}$ are gain and time constant, respectively. However, gain and time constant for the governor system is denoted by $G_{Gov}$ and $T_{Gov}$, respectively.

### Battery based energy storage (BBES)

Battery based energy resources is incorporated for immediate storage in case of excess power generation. The equivallent transfer function representation is depicted in (6).

6 $$\begin{aligned} H_{BBES}(s)=\frac{G_{BBES}}{1+sT_{BBES}} \end{aligned}$$

In (6), notations for the representation of gain and time constant of BBES are written as, $G_{BBES}$ and $T_{BBES}$, respectively.

### Airport microgrid system dynamics

System dynamics of AP${\mu }G_{d}$ system including inertia and damping, is written in (7).

7 $$\begin{aligned} H_{AP{\mu }G_{d}}(s)=\frac{1}{\xi +s\gamma } \end{aligned}$$

As given in (7), damping is depicted as $\xi$ whereas, for inertia of AP${\mu }G_{d}$, notation $\gamma$ is exploited.

### Block diagram representation of airport microgrid

**Fig. 2:**
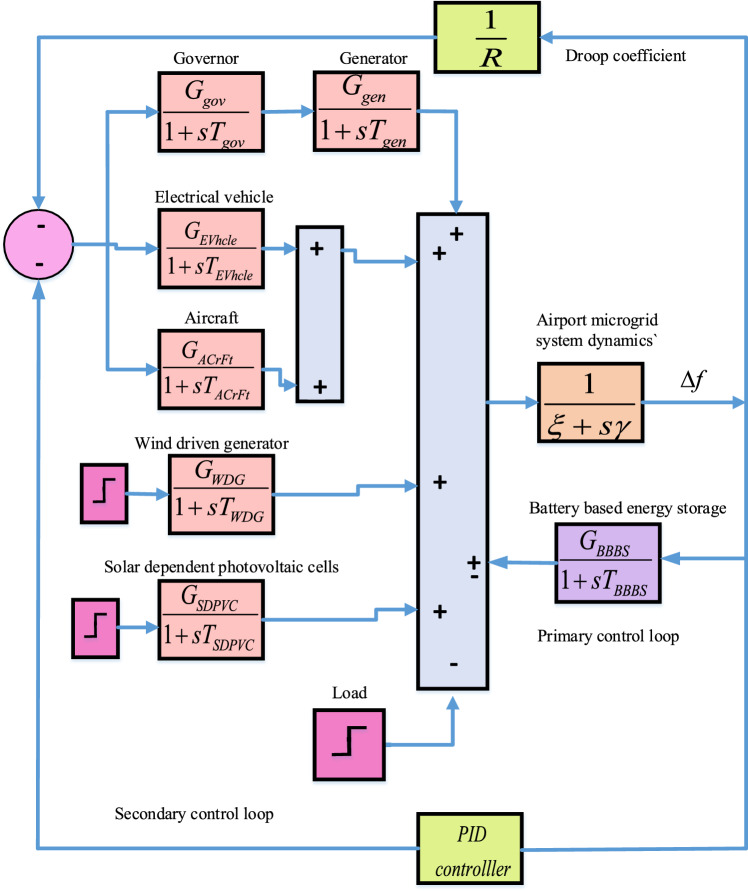
Architecture of AP${\mu }G_{d}$ System.

Utilizing the mathematical model generating and storage components, a detailed block diagram for considered AP${\mu }G_{d}$ is shown in Fig. 2. All components are represented through their corresponding transfer functions. As shown in Fig. 2, two control loops (primary and secondary) are categorized for specific purpose. The primary control loop is comprises of storage element (BBES), while distributed generating units except WDG and SDPVC, are utilized through secondary control loops. Both SDPVC and WDG are completely dependent to the nature, thus provide variable output. Output of WDG and SDPVC is of varying in nature hence creates unbalance between source and load. Due to this, the effect of WDG and SDPVC for the AP${\mu }G_{d}$ is taken in terms of disturbance. Additionally, droop coefficient, which is represented as 1/*R* incorporated in Fig. 2, which is crucial in controlling system frequency.

In order to simplify study, a number of assumptions are frequently used while modeling an Airport Microgrid ($AP \mu Gd$). These assumptions include linearized system dynamics, constant load profiles, ideal inverter responses, and the disgraded response for communication delays or high-frequency switching effects. Although these presumptions simplify controller design, they may reduce simulation realism. When applied to real-world situations, such simplifications may result in disparities in PID controller performance, particularly when highly variable renewable energy profiles, such as solar and wind, are present. Assuming a constant or slowly changing input could result in a PID design that is not responsive enough to sudden changes in power output, which could cause greater overshoot or deteriorate frequency stability.

**Table 2:** Components of Airport microgrid transfer function and their value^22,23^.

Component	Transfer function	Parameters
Solar dependent photovoltaic cells (SDPVC)	$H_{SDPVC}(s)=\frac{G_{SDPVC}}{1+sT_{SDPVC}}$	$T_{SDPVC}=1.8$, $G_{SDPVC}=1$
Wind driven generator (WDG)	$H_{WDG}(s)=\frac{G_{WDG}}{1+sT_{WDG}}$	$T_{WDG}=1.5$, $G_{WDG}=1$
Diesel operated generator (DOG)	$H_{DOG}(s)= \left( \frac{G_{Gen}}{1+sT_{Gen}}\right) \left( \frac{D_{Gov}}{1+sT_{Gov}}\right)$	$T_{Gen}=0.1$, $G_{Gen}=1$, $T_{Gov}=0.08$, $G_{Gov}=1$
Aircraft (ACrFt)	$H_{ACrFt}(s)\approx \frac{G_{ACrFt}}{1+sT_{ACrFt}}$	$T_{ACrFt}=1$, $D_{ACr}=1$
Battery based energy storage (BBES)	$H_{BBES}(s)=\frac{D_{BBES}}{1+sT_{BBES}}$	$T_{BBES}=0.1$, $K_{BBES}=1$
Electric vehicles (EVhcle)	$H_{EVhcle}(s)\approx \frac{G_{EVhcle}}{1+sT_{EVhcle}}$	$T_{EVhcle}=0.1$, $G_{EVhcle}=1$
Airport microgrid system dynamics (AP${\mu }G_{d}$)	$M_{A\mu Gd}(s)=\frac{1}{\xi +s\Upsilon }$	$\xi =0.012$, $\Upsilon =0.2$

### Overall transfer function for airport microgrid

The AP${\mu }G_{d}$ system involves multiple generating and storage components incorporated for power supply applications. Combining the transfer function of each components, an OATrFn for the whole system is derived. All individual FOTrFns are combined for the derivation of OATrFn.

**Fig. 3:**
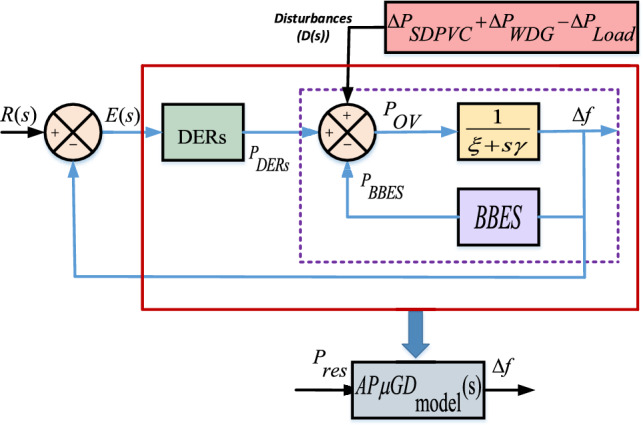
Equivalent model representation of AP${\mu }G_{d}$.

FOTrFn models for each unit are depicted with the considered parametric values in the Table 2 .

The OATrFn of $q^{th}$ order for an AP${\mu }G_{d}$ system in general form is given in (8).

8 $$\begin{aligned} H_{OV}(s)= \frac{\vartheta _{0}+\vartheta _{1}s+\vartheta _{2}s^{2}+\ldots +\vartheta _{q}s^{q-1}}{\theta _{0}+\theta _{1}s+\theta _{2}s^{2}+\ldots +\theta _{q}s^{q}} \end{aligned}$$

For the OATrFn of $q^{th}$ order given in (8), $\vartheta _i$ and $\theta _i$ are the coefficients for numerator and denominator, respectively. For $\vartheta _i$, expended values of *i* can be $i = 0,1,2,3 \dots , q-1$ whereas for $\theta _i$, *i* can be expended as $i = 0,1,2,3 \dots q$.

For a higher order system, analytical study and controller designing is typical and complex. Approximation of the higher order system into lower order model could be a feasible solution in order to perform analytical study and controller designing further. For the given generalized system, a first order plus delay time model (FOPDT) is obtained. Based on that, controller parameters are tuned further.

## First order plus delay time model

For a system of higher order, analysis and controller design is one of the most typical tasks due to its complexity. This becomes comparatively easier for analysis if a lower order model is obtained which replicate the behavior of the original system. An step response based reaction curve method, is utilized for deriving the FOPDT model^24^. This FOPDT model is further utilized for tuning of the PID controller.

**Fig. 4:**
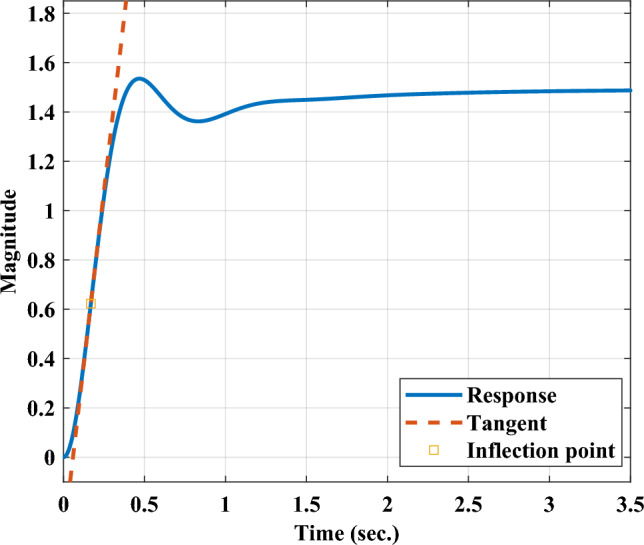
Process reaction curve method^23^.

Reaction curve for the AP${\mu }G_{d}$ system is shown in the Fig. 4. Based on that, parameters gain, time delay and time constant are obtained in order to derive the FOPDT model as given in (9).

9 $$\begin{aligned} M_{FOPDT}=\frac{G}{1+sT}e^{-sW} \end{aligned}$$

In (9), gain and time delay are denoted by *G* and *W* respectively, whereas time constant is represented by *T*. In next, Parameters *G*, *W* and *T* are calculated for the implementation of PID controller.

## PID controller implementation

To ensure uninterrupted supply to the load while maintaining the stability, a ${\mu }G_{d}$ must be facilitated with appropriate control mechanism. Several contributions for the designing and implementation of suitable control scheme utilising PID controller are given in^25–31^.

**Fig. 5:**
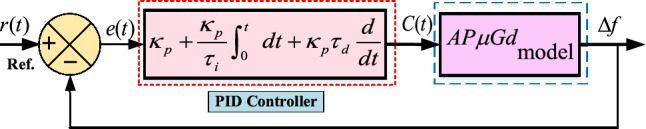
Close loop block diagram representation of AP${\mu }G_{d}$ model with PID Controller.

PID controller is well known controller, preferred for wide area of engineering applications. Significant contribution by researchers are reported in literature for the implementation of PID controller for various applications. Rathore et al. ^32^, discussed the PID control scheme for the positional controlling of the servo motor based on the luus-jaakola optimization approach. Further, PID controller is utilized for Doha water treatment plant utilizing a self learning based salp swarm optimization^33^.

### Structure of PID controller

**Fig. 6:**
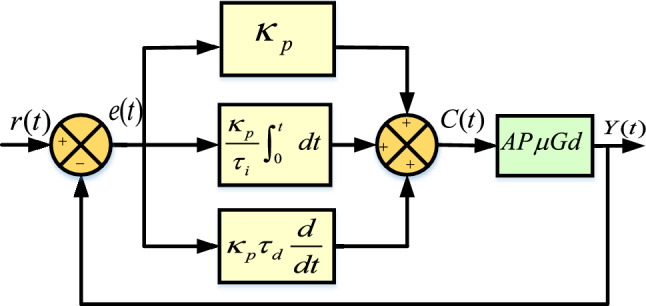
PID controller design.

A block diagram representation for the functioning of PID controller is depicted in Fig. 6. The controller consists of proportional, integral, and derivative components along with input, error signals, gains, and output, as formulated in (10).

10 $$\begin{aligned} C(t)=\kappa _pe(t)+\frac{\kappa _p}{\tau _i}\int _{0}^{t}e(t)dt+\kappa _p\tau _d\frac{de(t)}{dt} \end{aligned}$$

In (10), sequentially, proportional and integral components are formulated with corresponding constants $\kappa _p$ and $\tau _i$, respectively. In the last segment of (10), derivative component is formulated with constant $\tau _d$. To tune these constants, error minimization based approach i.e. integral absolute error (IAE) is utilised.

### IAE tuning methodology

To have a smoother response and minimum PID control efforts, error-index i.e. integral absolute error (IAE) is preferred among other error indices. IAE places equal importance on all errors, regardless of their magnitude. IAE index is applied for tuning the gain of PID controller. The mathematical formulation of IAE is depicted in (11) for the calculation of PID controller constants.

11 $$\begin{aligned} IAE=\int _{0}^{t}|e(t)|dt \end{aligned}$$

Utilizing the IAE and FOPDT model parameters (*G*, *W* and *T*), tuning rules for PID constants are formulated in (12), (13), and (14).

12 $$\begin{aligned} \kappa _p= &   \frac{0.65}{G}\left( \frac{W}{T} \right) ^{^{-1.044}} \end{aligned}$$

13 $$\begin{aligned} \tau _i= &   \frac{T}{0.9895+0.9539\left( \frac{W}{T} \right) } \end{aligned}$$

14 $$\begin{aligned} \tau _d= &   {0.508T}\left( \frac{W}{T} \right) ^{^{1.084}} \end{aligned}$$

## Overall power balance and frequency deviation

Power delivered from the generating and storage components are utilised for the load at the AP locality. Power balanced equation is given in (15).

15 $$\begin{aligned} P_{res} = P_{ov} - P_{L} \end{aligned}$$

The notations mentioned in the (15) are termed as residual power $(P_{res})$, load $(P_{L})$ and overall power $(P_{ov})$. As mentioned in Eq. (15), The gap between $P_{ov}$ and $P_{L}$ is defined by $P_{res}$. Equation (16) is exploited for obtaining $P_{ov}$ by summing each integrated generating and storage components algebraically.

16 $$\begin{aligned} P_{OV} = P_{SDPVC}+P_{WDG}\pm P_{BBES}+P_{ACrFt}+P_{DOG}+P_{EVhcle} \end{aligned}$$

The demand at the consumer terminal must be followed closely by the generation with minimum time delay in order to maintain the stable operation. The relation between $\Delta f$ and $P_{res}$ is given in Eq. (17).

17 $$\begin{aligned} \Delta f=\frac{{1}}{\xi s+\gamma }P_{res} \end{aligned}$$

As shown in Eq. (17), the relation of $\Delta f$ with $P_{res}$ is of direct proportionality. To maintain $\Delta f$ minimally, $P_{res}$ is required to maintain as minimum as possible. In other words, optimum fulfillment of load demand should be followed by the generation and storage units.

## Results and discussion

This section involves the graphical and tabular analysis of the overall AP${\mu }G_{d}$ system prior and later to PID controller implementation. Firstly, a combined transfer function is derived by incorporating each component of the AP${\mu }G_{d}$ system. To maintain the frequency profile, an efficient PID controller is designed. Gains are tuned to design an efficient PID controller.

### Transfer function for AP${\mu }G_{d}$ system

The block diagram architecture shown in Fig. 2 consisting the components of AP${\mu }G_{d}$, is utilized to derive the equivalent transfer function for the AP${\mu }G_{d}$ system in generalized form as depicted in (8) . The derived transfer function for the AP${\mu }G_{d}$ system is given in (18).

18 $$\begin{aligned} \begin{aligned} K_{over}=\dfrac{0.00088s^4+0.0402s^3 + 0.57s^2 +2.86s+3}{\begin{array}{c} {0.000016 s^6 + 0.000697 s^5 + 0.01262 s^4}\\ { + 0.1277s^3 +0.7772 s^2 + 2.45 s + 2.012 } \end{array}} \end{aligned} \end{aligned}$$

Further, analysis of the higher order OATrFn for AP${\mu }G_{d}$ system given in (18), is difficult and complex. To overcome the challenges associated with a higher order system, FOPDT model is derived utilizing reaction curve method. In Fig. 4, reaction curve for the AP${\mu }G_{d}$ system is shown consisting of system response, tangent line, and point of inflection. Based on the reaction curve, FOPDT parameters are obtained and exploited for deriving the FOPDT model. Exploiting generalized expression of FOPDT model shown in (9) for sixth order AP${\mu }G_{d}$ transfer function given in (18), the derived FOPDT model is depicted in (19).

19 $$\begin{aligned} \begin{aligned} K_{FOPTD}=\frac{1.4878}{1+0.2637s}e^{-0.0588s} \end{aligned} \end{aligned}$$

### AP${\mu }G_{d}$ system validation with approximated model

**Fig. 7:**
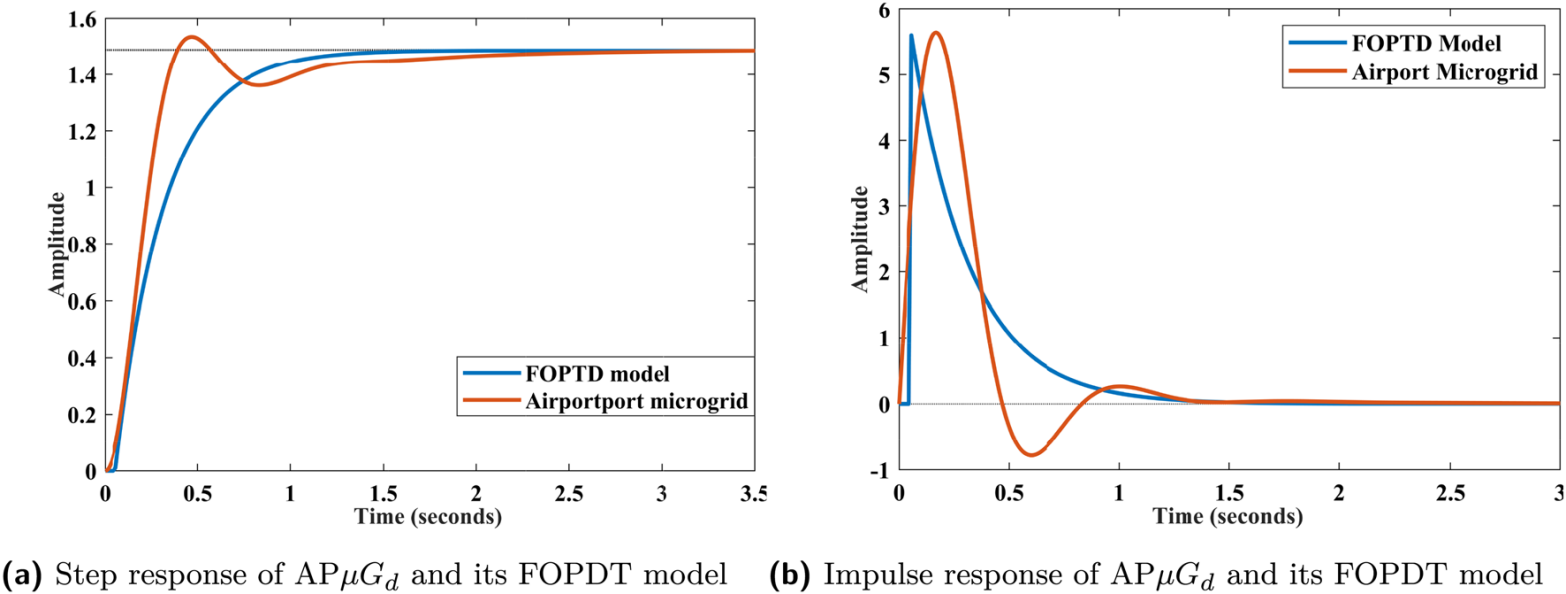
Step and impulse response of airport and FOPDT model.

**Fig. 8:**
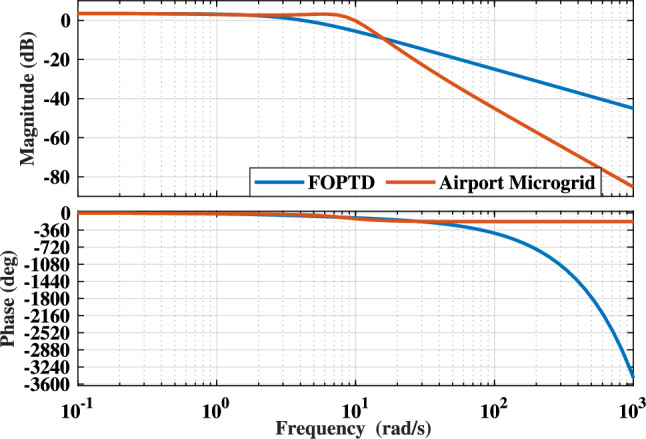
Bode plot of AP${\mu }G_{d}$ and its FOPDT model.

Deriving a model for an original system must require validation in order to verify the responses of model in replication with the original system. FOPDT model obtained for the AP${\mu }G_{d}$ system is verified through the step, impulse, and Bode plots given in Figs. 7a, b and 8, respectively. The Bode plot depicted in Fig.8, compares the frequency response of AP${\mu }G_{d}$ and its corresponding FOPDT approximation. The FOPDT model and the AP${\mu }G_{d}$ both exhibit comparable gain at low frequencies, which is usually more important for control reasons. From the responses, it is observed that the plots for the FOPDT model is closely following the responses of the original AP${\mu }G_{d}$ system. Time domain specifications are also tabulated for the AP${\mu }G_{d}$ system and FOPDT model, as mentioned in Table 3. AP${\mu }G_{d}$ system and its FOPDT model both have the peak value of 1.5351 and 1.4870, respectively. However, actual system and FOPDT model have no undershoot, but overshoot of magnitude 2.9549 is observed in case of AP${\mu }G_{d}$ system. Rise time for the AP${\mu }G_{d}$ system is noted as 0.2493 sec which is approx to the FOPDT model’s rise time, having the value of 0.5794. Both AP${\mu }G_{d}$ system and its FOPDT model are observed as stable with settling time 1.8304 sec and 1.0904 sec, respectively. The peak time of step response of the AP${\mu }G_{d}$ system is observed as 0.4711 sec whereas, peak time for FOPDT model’s step response is noted as 2.0523 sec. Moreover, Figs. 7a, b and 8 are qualitatively sufficient whereas the data tabulated in Table 3 assure the validation of the FOPDT model against the AP${\mu }G_{d}$ system. Fig.8, is provided to validate the AP${\mu }G_{d}$ model in frequency domain. As FOPDT model is verified, it is further utilized for the calculation of the PID controller parameters as per the Eq. (12), (13), and (14).

Transient response indications, particularly overshoot and settling time, are crucial in an AP${\mu }G_{d}$ system. The degree to which a frequency momentarily surpasses its intended setpoint in reaction to disruptions or control measures is known as overshoot. Overshoot of magnitude ($2.9549\%$) can have a major impact on microgrid stability since it can cause component stress, activate protection mechanisms, or even cause instability throughout the system, especially in systems with sensitive loads and tightly coupled renewable sources. It may lead to losss of synchronization among various components of the AP${\mu }G_{d}$ system.

**Table 3:** Time domain specifications.

	Time domain specifications					
	Rise time (sec)	Settling time (sec)	Peak	Peak time (sec)	Overshoot (%)	Undershoot (%)
AP${\mu }G_{d}$	0.2493	1.8304	1.5351	0.4711	2.9549	0
FOPDT model	0.5794	1.0904	1.4870	2.0523	0	0

**Fig. 9:**
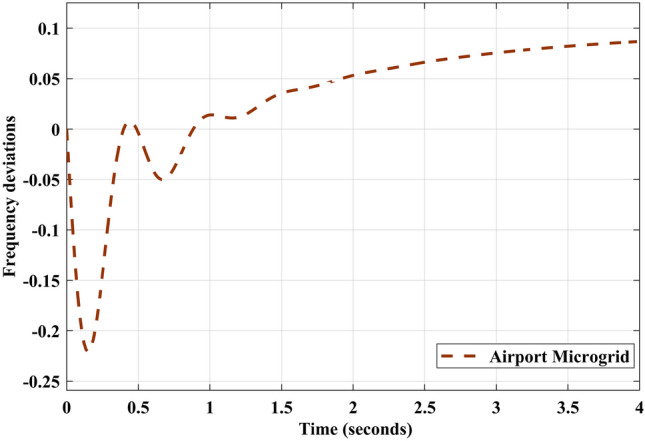
Frequency response of AP${\mu }G_{d}$ system without control strategy.

### AP${\mu }G_{d}$ system analysis with controller implementation.

**Fig. 10:**
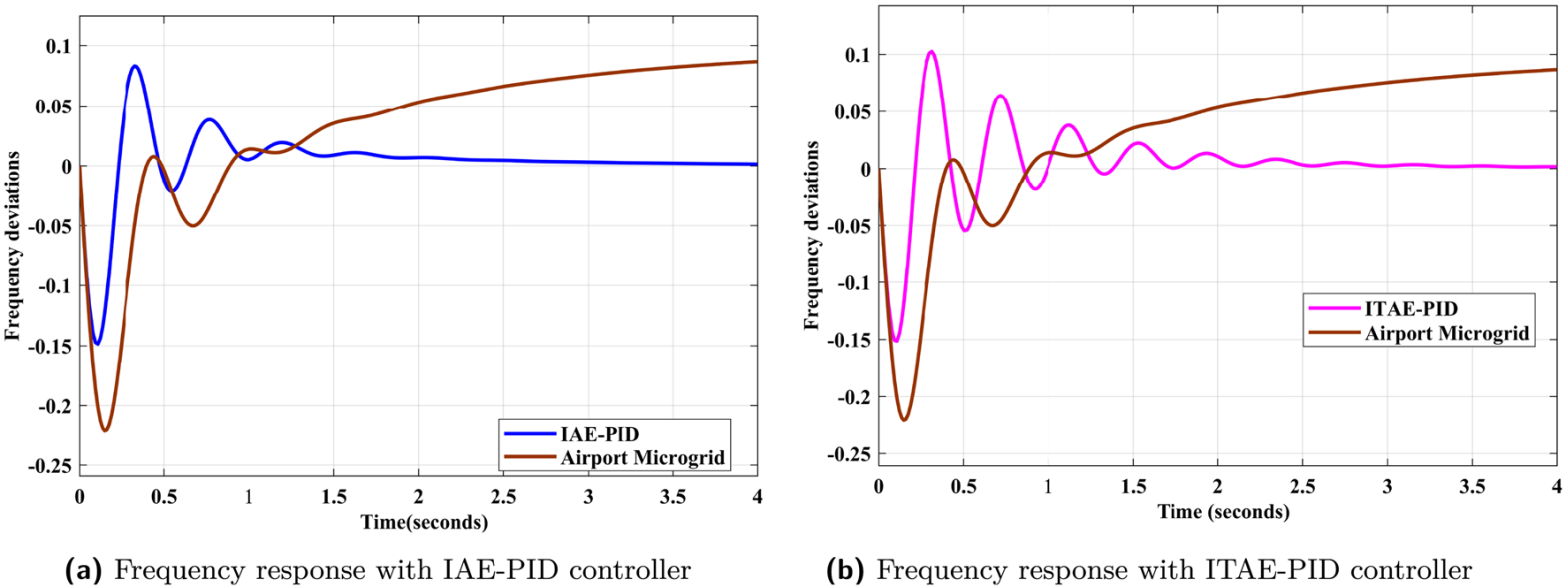
Frequency response of airport microgrid with PID controller.

**Fig. 11:**
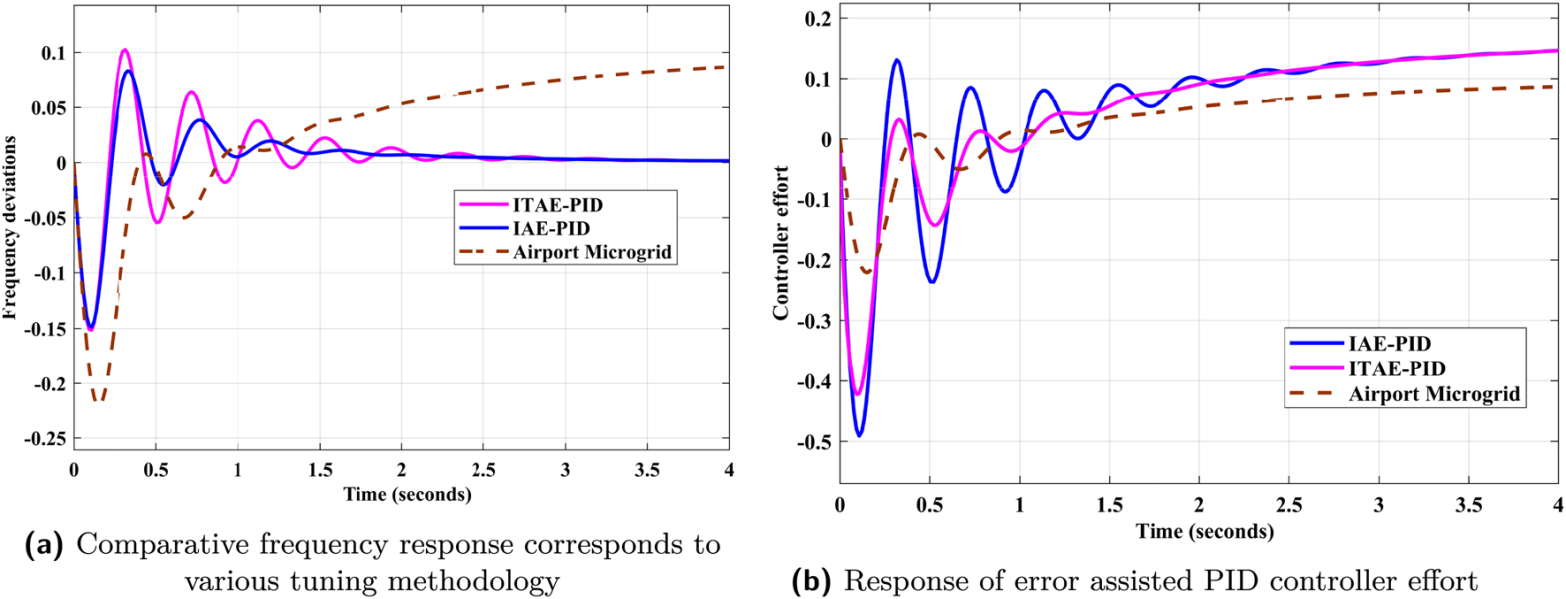
Comparison of frequency response with PID controller and their control efforts.

Freequency response of AP${\mu }G_{d}$ system is depicted in Fig. 9. It shows that frequency deviations of AP${\mu }G_{d}$ is increasing as time increases. It violets the balance between generation and loads. It should be maintained in such a way that frequency deviation must be zero. To achieve this, PID controller is utilized in this contribution. PID controller for frequency deviation are tuned using IAE error index. For comparative analysis, ITAE tuned PID controller is also designed and implemented. The effectiveness of the controller utilizing IAE and ITAE indices are validated through the quantitative and qualitative data. To analyze the qualitative aspects of the control design, frequency deviation responses with and without controller implementation are plotted Fig. 10b and a. In Fig. 10b, ITAE tuned PID controller eliminates the error exits in AP${\mu }G_{d}$ frequency response and maintains frequency deviations to zero. But in Fig. 10a, IAE tuned PID controller eliminates the error and maintains power balance between generation and load by keeping deviation to zero. The frequency response depicted in Fig. 11a, concludes that response obtained from IAE tuned PID controller is comparatively better than ITAE tuned PID controller, as it is less oscillatory. Further, quantitative aspects are also analyzed through tabular data of time domain specification and error indices corresponding to AP${\mu }G_{d}$ system.

**Table 4:** PID controller parameters using error indices method.

Tuning method	$\kappa _p$	$\tau _i$	$\tau _d$
IAE	2.0930	9.5421	0.0551
ITAE^34^	2.5337	9.6512	0.0249

As given in Table 4, calculated controller parameters utilising IAE indice based formulaes given in Eqs. (12), (13) and (14), are shown. It is observed that $k_p$ for the IAE indices is smaller as compare to the ITAE indices having the values 2.0930 and 2.5337 respectively. The calculation, results the values for $T_i$ utilising ITAE and IAE indices are 9.6512 and 9.5421 respectively. In case of $T_d$ parametric values are tabulated as 0.0551 and 0.0249 based on IAE and ITAE error indices respectively. These paramemeters are further utilized to tune the PID controller.

To maintain frequency deviation within the limit for maintaining stability, PID controller with IAE amd ITAE error indices, is implemented as shown in (21) and (20), respectively. Table 4, consists of values for controller parameter $\kappa _p$, $\kappa _i$ and $\kappa _d$.

20 $$\begin{aligned} K_{IAE-PID}= &   2.0930+\frac{9.5421}{s}+ 0.0551s \end{aligned}$$

21 $$\begin{aligned} K_{ITAE-PID}= &   2.5337+\frac{9.6512}{s}+ 0.0249s \end{aligned}$$

**Fig. 12:**
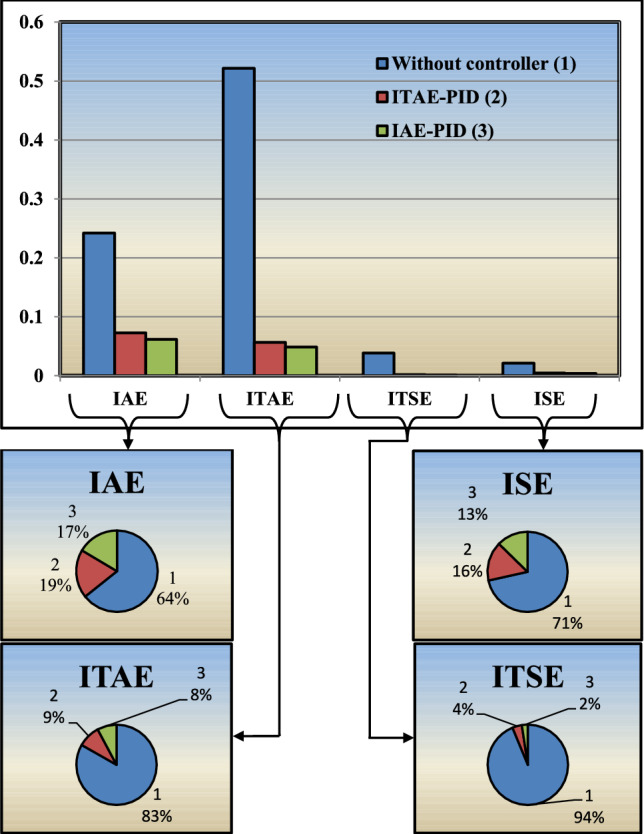
Bar graph of error indices for system with and without controller.

Frequency deviation prior and after the implementation of IAE driven PID controller is analyzed as shown in 9 and 10a respectively. From the responses, behavior of system can be clearly observed that with controller implementation, frequency deviation is greatly minimized compare to prior. To verify the potency of IAE driven PID controller, comparative analysis is performed by comparing the results with ITAE assisted PID controller. Frequency deviation responses for system versus both indices driven controllers, are plotted in Figs. 10a and 11a. In Fig. 11b, frequency deviation plots for the A$\mu$Gd, with controller implementation using IAE and ITAE error indices are plotted on the same scale for comparative analysis. Step response for ITAE driven controller for minimization of frequency deviation has overshoot more than IAE based PID controller. For the analysis of controller performance, control effort response for both techniques are compared with the response of the original A$\mu$Gd system. Error index in terms of frequency deviation are plotted for the system under three scenarios i.e. system without controller implementation, ITAE based PID controller implementation and IAE assisted PID controller implementation. Error is calculated in absolute and squared form. Both absolute and squared error are integrated with time. Table 6 consists of error index (IAE, ITAE, ISE, and ITSE), for all three scenarios. From the Table 6, performance of IAE based PID controller can be evaluated as the error index corresponding to the said controller appeared minimum compare to other approach. A bar graph is plotted in Fig. 12 for the qualitative analysis of IAE based PID controller compare to other utilized approach. In Fig. 12, error index in numerical and percentage based representation are plotted in in bar graph and corresponding pie charts, respectively. In case of IAE assisted PID controller, IAE and ISE error index are observed as minimum with the 17% and 13% receptively. While integrated with the time, the percentage participation of error index ITAE and ITSE is calculated as 8% and 2%, respectively, which is minimum compare to others.

The potency of implemented control technique, is well validated through the verification of adequate graphical and tabular data.

**Table 5:** Time domain specifications corresponding to tuning rules.

Method	Time domain specifications					
	Rise time (sec)	Settling time (sec)	Peak	Peak time (sec)	Overshoot (%)	Undershoot (%)
Without control	0.0698	0.5369	1.2544	0.1786	25.4408	0
IAE-PID	0.1758	2.0267	1.4594	0.4385	45.9374	0
ITAE-PID^34^	0.5883	6.8357	0.9984	21.7943	0	0

**Table 6:** Tabular comparison of different error indices.

	Error indices			
	IAE	ITAE	ITSE	ISE
Without controller	0.2418	0.5218	0.03841	0.02107
IAE-PID	0.0619	0.04881	0.0009927	0.003733
ITAE-PID^34^	0.07242	0.05633	0.001568	0.004661

Implementing a PID controller provides a practical and affordable way to ensure frequency stability and system reliability in an actual Airport Microgrid ($AP \mu Gd$) arrangement. Because PID controllers require little in the way of hardware and processing, they can be integrated with digital control platforms or existing programmable logic controllers (PLCs) without requiring costly updates. Because of their simplicity, they are perfect for airport infrastructure where operational continuity is crucial because they lower design and maintenance costs. Additionally, engineers’ extensive expertise with PID control speeds up implementation and troubleshooting. Practical implementation, however, must take into account adjusting robustness under changing load conditions and renewable energy intermittency. To guarantee optimal performance throughout operational scenarios, adaptive or gain-scheduled variations may be necessary.

## Conclusion

This study effectively addresses the issues of frequency regulation in Airport Microgrid (AP${\mu }G_{d}$ with aircraft integration. To achieve desired frequency stability, an integral absolute error (IAE) assisted proportional-integral-derivative (PID) controller is employed. The modeling of AP${\mu }G_{d}$ is carried out considering the gain and time constant of the corresponding components, which are combined further for deriving the overall transfer function (OATrFn). The derived OATrFn of AP${\mu }G_{d}$ is of higher order, due to this, analysing the system efficiently is tedious and time consuming process. To overcome this issue, a First-Order Plus Delay Time (FOPDT) model is used to approximate the higher-order OATrFn to overcome the difficulties in assessing and implementing an efficient controller. Further, FOPDT model and IAE error indice were used to effectively adjust the PID controller gains.

The system’s response is analyzed both with and without the PID controller, highlighting the controller’s efficacy in mitigating frequency deviations. Time and frequency domain specifications were critically assessed through tabular data, and the results, presented as plots and tables, validated the improved response of the system post-controller implementation. These findings demonstrate the significant potential of IAE assisted PID controller in enhancing microgrid stability.

Future work of this article may explore real-time hardware-in-the-loop validation, economic feasibility studies, and robustness testing under extreme renewable fluctuations. Moreover, PID control designs utilizing alternative error indices to optimize performance may be implemented.

## Data Availability

No datasets were generated or analysed during the current study.
